# The National Prevention Conference’s prevention report – the development of a concept for the 2019 report

**DOI:** 10.17886/RKI-GBE-2018-052

**Published:** 2018-05-16

**Authors:** Stefanie Liedtke, Stefan Gravemeyer, Guy Oscar Kamga Wambo

**Affiliations:** 1 German National Association of Statutory Health Insurance Funds, Department of Health; 2 German Social Accident Insurance (DGUV), Department of Safety and Health; 3 German Statutory Pension Insurance Scheme, Social Medicine and Rehabilitation Division

The National Prevention Conference (NPC) has been tasked with delivering the first ever cross-institutional prevention report by 1 July 2019. The report will include an initial assessment of the implementation of the NPC’s Federal Framework Recommendations on health promotion and disease prevention in people’s living and working environments [1]. In February 2017, the NPC agreed upon a draft concept for the report. The elaboration of a more detailed concept as well as its methodological implementation, starting in spring 2018, will be carried out with support of the IGES Institute.

In line with section 20d (4) of Book 5 of the German Social Code, the prevention report will focus on describing the measures implemented to meet the objectives and reach the target groups set out in the Federal Framework Recommendations, NPC stakeholders’ experiences gained through cooperation and quality assurance in this context, and stakeholders’ expenses. Results from the health monitoring conducted at the Robert Koch Institute and the federal state level will be incorporated as well. Another focus will be on illustrating the current state of research regarding the effectiveness of disease prevention and health promotion measures related to people’s living and working environments. In addition, the report will cover the reduction of social and gender disparities in health care opportunities – a task that needs to be undertaken in society as a whole – as an inter-disciplinary issue.

The institutions involved in the NPC and their member organisations will use the results to refine their activities in the field of promotion of health, safety, participation and disease prevention. This will also include examining options to improve documentation and evaluation. Finally, the report will also seek to identify starting points for refining shared goals and expanding cooperation and coordination within the context of the national prevention strategy as laid out in section 20d of Book 5 of the German Social Code.
